# DEP-track: a motion-aware framework for large-scale cell tracking and crossover frequency estimation in dielectrophoresis

**DOI:** 10.3389/fbinf.2026.1821804

**Published:** 2026-04-23

**Authors:** Sena Lee, Seungyeop Choi, Yerin Lee, Hyunmin Bae, Junghun Han, Yoon Suk Kim, Sang Woo Lee, Sejung Yang

**Affiliations:** 1 Department of Precision Medicine, Wonju College of Medicine, Wonju, Republic of Korea; 2 Department of Biomedical Engineering, Yonsei University, Wonju, Republic of Korea; 3 Institute of Global Health Technology (IGHT), Korea University, Seoul, Republic of Korea; 4 Department of Biomedical Laboratory Science, Wonju College of Medicine, Yonsei University, Wonju, Republic of Korea; 5 Department of Medical Informatics and Biostatistics, Wonju College of Medicine, Wonju, Republic of Korea

**Keywords:** cell tracking, crossover frequency, dielectric phenotyping, dielectrophoresis, single-cell analysis, time-lapse imaging

## Abstract

Precise and scalable analysis of single-cell responses under dielectrophoresis (DEP) remains challenging, particularly in long-term experiments involving frequency modulation and dense cell populations. Conventional DEP workflows rely heavily on manual trajectory inspection or repeated measurements, limiting throughput, reproducibility, and statistical power. Here, we present DEP-Track, a motion-aware computational framework designed for automated large-scale trajectory preservation and crossover frequency estimation from frequency-modulated DEP microscopy data, where the crossover frequency is defined as the point at which the direction of DEP-induced cell motion reverses. The framework integrates anchor-free cell detection with motion-aware trajectory association to maintain single-cell identity across abrupt polarity-induced motion transitions over tens of thousands of frames. By unifying velocity-based estimation under fixed frequencies and trajectory-based estimation under continuous frequency modulation, DEP-Track enables automated extraction of statistically consistent estimates of crossover frequency at the single-cell level from repeated crossover events within a single experiment. In long-term time-lapse imaging experiments (13,200 frames), hundreds of cells were continuously tracked, enabling population-scale analysis without repeated experimental runs. Importantly, this study focuses exclusively on estimating the crossover frequency at the single-cell level. The estimated crossover frequencies showed strong agreement with conventional analysis workflows and previously reported measurements, confirming analytical accuracy and reproducibility. By transforming DEP analysis into a scalable and reproducible computational workflow, DEP-Track establishes a framework for high-throughput dielectric phenotyping based on crossover frequency.

## 1 Introduction

**FIGURE 1:**
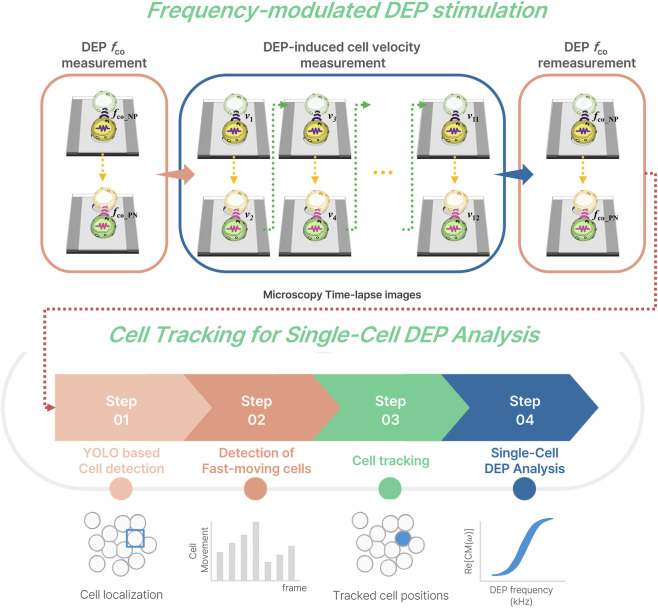
Overview of the frequency-modulated DEP stimulation and single-cell tracking framework.

Dielectrophoresis (DEP) is a phenomenon in which neutral but polarizable particles experience motion in non-uniform electric fields (Pohl et al., 1978). Because DEP enables label-free and non-invasive manipulation of cells based on intrinsic dielectric properties, it has become a powerful platform for biomedical and biotechnological applications. DEP has been widely used for cancer diagnostics through crossover frequency ( $f_{co}$ ) discrimination (Varmazyari et al., 2022; Nguyen et al., 2024; Uddin and Chen, 2024), real-time monitoring of cellular drug responses (Jeong et al., 2018; Tehrani et al., 2025), and microfluidic cell sorting of heterogeneous populations (Gao et al., 2024; Najafipour et al., 2025). These applications highlight the potential of DEP as a quantitative tool for probing single-cell dielectric behavior (Julius et al., 2024; Tian et al., 2024; Mondal et al., 2025). In recent years, DEP systems have evolved toward higher throughput, dynamic frequency modulation, and automated microfluidic integration (Yao et al., 2024). Modern DEP platforms allow simultaneous observation of large cell populations under precisely controlled and continuously varying electric fields. Correspondingly, image-based analysis has progressed from qualitative observation of cell displacement to quantitative extraction of velocity, trajectory, and crossover frequency, enabling more detailed investigation of DEP-driven cellular motion dynamics. However, robust trajectory preservation in long-term, frequency-modulated DEP experiments remains technically challenging (Choi et al., 2018; Choi et al., 2020; Maška et al., 2023). DEP microscopy images often exhibit low contrast and structured electrode backgrounds, which complicate accurate cell localization (Chai et al., 2024). Moreover, frequency modulation can induce transitions between positive and negative DEP regimes, leading to abrupt changes in force direction and highly non-stationary, nonlinear cell trajectories. Unlike conventional time-lapse microscopy experiments characterized by relatively smooth endogenous cellular motion, frequency-modulated DEP introduces externally driven polarity shifts that fundamentally alter trajectory dynamics. These motion patterns challenge assumptions commonly adopted in conventional frame-to-frame tracking algorithms. The challenge is further amplified in dense cell populations, where identity ambiguity and partial overlap frequently occur. Long-duration experiments spanning thousands to tens of thousands of frames increasingly necessitate automated tracking strategies capable of preserving cell identity with minimal manual correction and parameter tuning. In addition to imaging-based DEP analysis, several established platforms have enabled automated and high-throughput characterization of dielectrophoretic responses. Commercial systems such as 3DEP (DEPtech), developed based on the work of Hughes and colleagues, measure DEP responses of cell populations across multiple frequencies and estimate dielectric parameters such as membrane capacitance and cytoplasmic conductivity through spectrum fitting (Hoettges et al., 2019). More recently, OpenDEP has been introduced as an open-source alternative that combines low-cost lab-on-a-chip hardware with image-based analysis to extract DEP spectra and perform dielectric modeling (Tivig et al., 2023). While these platforms provide efficient and automated DEP analysis, they primarily rely on population-level measurements and spectrum-based parameter estimation, and do not explicitly preserve long-term individual cell trajectories under dynamic frequency modulation. In contrast, trajectory-based imaging approaches enable direct analysis of single-cell motion dynamics under frequency-modulated DEP conditions. However, robust large-scale trajectory preservation and automated $f_{co}$ estimation from such data remain challenging, particularly in dense and long-duration experiments. By explicitly preserving single-cell trajectories over long time scales, the proposed framework enables direct extraction of $f_{co}$ distributions at the single-cell level from a single experiment, thereby reducing the need for repeated measurements and providing improved insight into cell-to-cell heterogeneity under dynamic DEP conditions. Existing DEP motion analysis approaches can be categorized into classical image processing methods, machine learning pipelines based on handcrafted features, and more recent deep learning–based detection frameworks. Early methods relied on thresholding (Voldman, 2006; Vaillier et al., 2016; Liu et al., 2020), centroid tracking, or optical flow, typically limited to tracking small numbers of cells and requiring repeated experiments for statistical analysis (Henslee et al., 2011; Gascoyne and Shim, 2014). Subsequent approaches incorporated multi-stage preprocessing and segmentation pipelines (Choi et al., 2020), improving robustness but remaining sensitive to parameter tuning and imaging variability. More recently, deep learning–based detection methods have significantly improved automation in DEP image analysis (Ajala et al., 2021). Recent studies have further expanded this direction by integrating multimodal DEP-imaging flow cytometry with high-speed optical tracking for single-cell phenotyping (Arzhang et al., 2024). However, these approaches primarily focus on detection accuracy and do not explicitly address long-term trajectory continuity under frequency-modulated DEP conditions. In parallel, broader advances in deep learning for microscopic cell segmentation and instance detection—including CNN- and transformer-based architectures—have provided powerful tools for improving robustness under low-contrast and structurally complex imaging conditions (Bhattiprolu, 2025; Wang et al., 2025). Many existing approaches primarily emphasize detection accuracy or short-term motion analysis, whereas the combined effects of frequency-modulated DEP stimulation, imaging variability, and dense cell interactions continue to challenge long-term trajectory continuity and identity preservation at scale. General-purpose multi-object tracking frameworks, such as Deep SORT (Wojke et al., 2017), BoT-SORT (Aharon et al., 2022), ByteTrack (Zhang et al., 2022), and OC-SORT (Cao et al., 2023), originally developed for computer vision applications, have also been explored in biomedical imaging contexts. However, these methods may introduce additional computational complexity when applied to dense, long-duration microscopy sequences, potentially limiting scalability in high-throughput frequency-modulated DEP experiments. In this study, we propose DEP-Track, a motion-aware computational framework designed to preserve single-cell identity under frequency-modulated DEP stimulation. Unlike conventional tracking approaches optimized for natural scene dynamics, DEP-Track explicitly accounts for abrupt polarity-induced motion transitions and structured electrode backgrounds in DEP microscopy, enabling robust trajectory continuity in dense and highly non-stationary conditions. In summary, this study makes two primary contributions. First, we introduce a large-scale tracking framework tailored for long-term, frequency-modulated DEP experiments, enabling robust preservation of single-cell identity across extended time sequences with reduced parameter sensitivity. Second, we establish an integrated DEP analysis pipeline that combines trajectory preservation with automated $f_{co}$ estimation, enabling population-level analysis of $f_{co}$ distributions from a single experiment without repeated measurements or manual post-processing. Unlike conventional spectrum-based approaches, this framework focuses on motion-derived estimation of $f_{co}$ at the single-cell level. An overview of the proposed detection–tracking–analysis pipeline is illustrated in Figure 1. This integrated framework provides a scalable and reproducible foundation for high-throughput dielectrophoresis analysis and dielectric phenotyping under dynamic DEP stimulation. This study focuses on estimating $f_{co}$ as a key descriptor of DEP-induced cellular behavior, without attempting to extract underlying dielectric parameters such as conductivity and permittivity.

## 2 Materials and methods

### 2.1 DEP experimental setup and image acquisition

Frequency-modulated dielectrophoresis (DEP) experiments were performed using a custom microfluidic DEP platform operated by a LabVIEW-based automated DEP system, consistent with previously reported configurations (Kim et al., 2017; Choi et al., 2020). The DEP microfluidic chip was based on a circular-hole electrode array fabricated on a SiO_2_ substrate. The electrode layer consisted of a 0.1 μm-thick chromium (Cr) film patterned by photolithography and lift-off, followed by deposition of a 0.8 μm-thick SiO_2_ insulating layer. Detailed chip fabrication procedures are consistent with previous reports (Choi et al., 2020; Park et al., 2020). An alternating current (AC) signal was delivered to the chip through a custom probe station (Modusystems, Hanam, Republic of Korea). The DEP system consisted of a control module, a signal generation module, and an observation module. The input signal was generated using an arbitrary function generator (NI PCI-5421, National Instruments, Austin, TX, USA), and the applied voltage amplitude and frequency were monitored using an oscilloscope (WaveSurfer 432, Teledyne LeCroy, Chestnut Ridge, NY, USA). The AC voltage amplitude was fixed at 2 V_p-p_, and the input frequency was modulated according to the experimental protocol described below. Prior to experiments, cells were harvested and resuspended in DEP buffer following standard preparation procedures. Human breast cancer MCF-7 cells were suspended in a low-conductivity DEP buffer composed of 8.6% (w/w) sucrose, 0.3% (w/w) D-glucose, 0.20% (v/v) phosphate-buffered saline (PBS), and 1.0 mg/mL bovine serum albumin (BSA). In addition to MCF-7 cells, other cell lines (HeLa, A549, and Jurkat) were evaluated under identical experimental and imaging conditions to assess the generalizability of the proposed framework, with the cell type being the only variable. The conductivity of the buffer was measured to be approximately 60 μS/cm. The initial cell concentration was adjusted to achieve approximately 100–500 cells within the field of view. Prior to DEP stimulation, the cell suspension was introduced into the microfluidic chip chamber. Bright-field time-lapse microscopy images were acquired using an inverted optical microscope coupled to a digital camera (Motionscope M3, Redlake, San Diego, CA, USA). Images were recorded at 10 frames per second as 8-bit grayscale images with a spatial resolution of 1,024 × 1,280 pixels, covering an approximately 1.5 mm^2^ field of view. Each experiment generated 13,200 consecutive frames, corresponding to 1,320 s of continuous DEP stimulation. To assess experimental reproducibility, three independent experiments were performed under identical DEP modulation protocols. Cells that exited the predefined valid observation region during acquisition were excluded from subsequent quantitative trajectory and $f_{co}$ analyses.

### 2.2 DEP stimulation protocol and dataset construction for tracking and crossover frequency estimation

**FIGURE 2:**
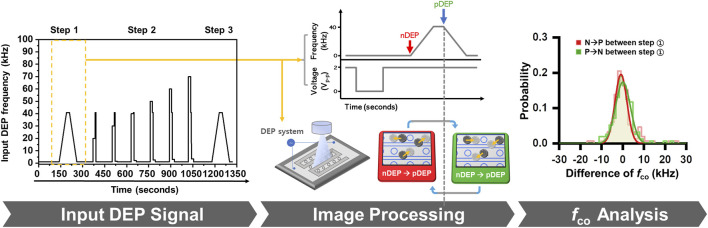
Frequency-sweeping DEP signal design and image-based $f_{co}$ analysis.

To evaluate the proposed tracking algorithm, we designed a three-step frequency modulation protocol for DEP $f_{co}$ estimation. This protocol builds upon previously reported DEP measurement procedures (Choi et al., 2018; Choi et al., 2020; Park et al., 2020), but is newly structured in this study to enable trajectory-based analysis of single-cell dynamics. In Step 1, the input frequency was continuously swept between 1 and 41 kHz at a rate of ±800 Hz/s to obtain an initial estimate of $f_{co}$ (Table 1; Figure 2). In Step 2, cells were exposed to multiple static-frequency DEP conditions to quantify DEP force-induced velocity under fixed electric-field regimes, which was used to refine the estimation of $f_{co}$ . In Step 3, the frequency sweep protocol was repeated to validate and improve the robustness of the measured $f_{co}$ . The cell radius (*r*_cell_), which affects DEP-induced motion and $f_{co}$ as described by the DEP force relationship (Park et al., 2020), was remeasured at each step to account for potential variations in cell state (Cadart et al., 2019; Urbanska et al., 2020). Using this protocol, cell movements on the DEP microfluidic chip were recorded as time-lapse microscopy image sequences. Each experiment was conducted for 1,320 s at 10 frames per second and repeated three times to ensure measurement reproducibility. Additional details of the DEP microfluidic system and cell handling procedures can be found in previous studies (Choi et al., 2020; Park et al., 2020). Multiple frequency sweeps and measurements were performed to repeatedly observe the same transition between positive DEP (pDEP) and negative DEP (nDEP), allowing estimation of a single $f_{co}$ for each cell. The acquired image sequences were processed using the proposed DEP-Track framework to reconstruct single-cell trajectories, from which displacement, velocity, and direction changes induced by frequency modulation were extracted. To train and evaluate the proposed framework, three large-scale DEP imaging datasets with varying cell densities were constructed (Table 2). The number of cells per frame ranged from approximately 100–500. For detector development, 45 microscopy images were manually annotated for training and validation. Although the number of annotated images is relatively small, each image contains a large number of cells (hundreds per frame), resulting in a substantial number of training instances. In addition, to mitigate overfitting and improve generalization, geometric data augmentation strategies were applied, including horizontal flip, vertical flip, and origin symmetry, taking into account the structured electrode patterns inherent to DEP microfluidic images. Mosaic augmentation was also employed to enhance detection robustness under complex backgrounds and to improve small-cell visibility (Takahashi et al., 2019). Detection testing was performed using 48 independent images sampled exclusively from the tracking datasets, ensuring that no training images were included in the test set. For trajectory reconstruction and $f_{co}$ estimation, three independent full-length time-lapse datasets (13,200 frames each; total 39,600 frames) were used exclusively for evaluation. This strict separation between training and evaluation datasets prevented data leakage and ensured an unbiased and reliable assessment of detection, tracking, and $f_{co}$ estimation performance.

**TABLE 1:** List of input DEP frequencies with AC 2 Vp-p.

Step	Trap frequency (kHz)	Input frequency (kHz)	Output parameter
1-(i)	1	1 to 41 (+800 Hz/s)	$r_{cell}$ *,* $f_{co}$
1-(a)	41	41 to 1 (−800 Hz/s)	
2-(i)	1	20	$r_{cell}$ *,* $f_{co}$
2-(a)	41	1.0	
2-(iii)	1	30	
2-(b)	41	1.5	
2-(v)	1	40	
2-(vi)	41	2.0	
2-(vii)	1	50	
2-(viii)	41	2.5	
2-(c)	1	60	
2-(x)	41	3.0	
2-(xi)	1	70	
2-(xii)	41	3.5	
3-(i)	1	1 to 41 (+800 Hz/s)	$r_{cell}$ *,* $f_{co}$
3-(a)	41	41 to 1 (−800 Hz/s)	

*In each step, the trap frequencies were set to 1 and 41 kHz, where the nDEP, and pDEP, forces were effectively induced, respectively. After identifying that most cells were trapped by the given frequency, each trap and test frequency in Table 2 was sequentially applied to measure cell DEP, mobility. Steps (a), (b), and (c) correspond to (Vassilvitskii and Arthur, 2006; Urbanska et al., 2020; Dendorfer et al., 2021), respectively.

**TABLE 2:** Dataset for multiple-cell tracking in imaging-based DEP experiments.

Average cell count per frame	Cell type	Detection		Tracking
		Train & validation	Test	
		Image	Image	Frame
100∼200	MCF-7	20	—	—
200∼300		—	16	13,200 (dataset 1)
300∼400		3	16	13,200 (dataset 2)
400∼500		22	16	13,200 (dataset 3)
Total		45	48	39,600

*Detection testing images were independently sampled from the Test 1 to Test 3 tracking datasets, with both nDEP, and pDEP, conditions intentionally included to cover a wide range of DEP, responses.

### 2.3 DEP-track tracking framework

**FIGURE 3:**
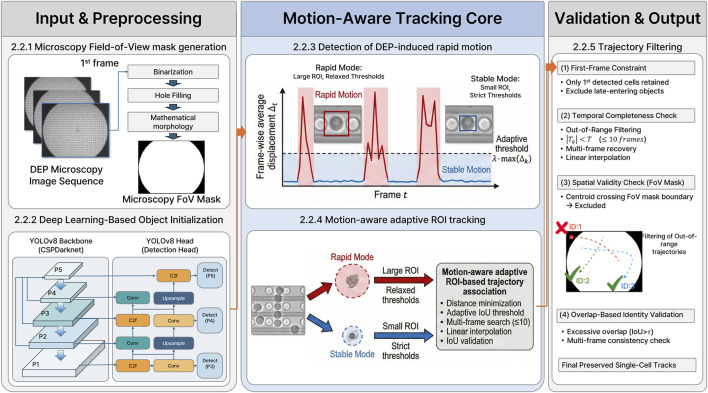
Overview of the DEP-Track framework for motion-aware single-cell tracking under frequency-modulated DEP. The framework is organized into three modules: Input & Preprocessing (left), Motion-Aware Tracking Core (center), and Validation & Output (right). In the preprocessing stage, a microscopy field-of-view (FoV) mask is generated from the first frame using binarization and morphological operations to define the valid observation region, followed by deep learning–based object detection (YOLOv8) for centroid–radius initialization. The tracking core identifies DEP-induced rapid-motion intervals by computing the frame-wise average displacement Δt and applying an adaptive threshold λ·max (Δk). Based on the detected motion regime (rapid or stable), motion-aware adaptive ROI tracking dynamically adjusts distance and IoU thresholds to preserve trajectory continuity. In the validation stage, reconstructed trajectories are evaluated through first-frame constraints, temporal completeness checks (|T_q| < T), spatial validity using the FoV mask, and overlap-based identity validation, yielding the final preserved single-cell tracks.

The proposed DEP-Track framework is designed to preserve single-cell identity under frequency-modulated DEP stimulation by explicitly accounting for abrupt motion transitions and structured imaging backgrounds. The overall pipeline consists of five sequential components: (1) Microscopy Field-of-View (FoV) mask generation, (2) deep learning-based object initialization, (3) DEP-induced rapid-motion identification, (4) motion-aware adaptive ROI tracking, and (5) out-of-range filtering and identity validation. The complete workflow is summarized in Figure 3.

#### 2.3.1 Microscopy field-of-view (FoV) mask generation

DEP microscopy images exhibit a circular field-of-view surrounded by peripheral regions affected by illumination distortion and electrode-induced artifacts. To ensure that trajectory analysis is restricted to the valid observation region, a microscopy field-of-view (FoV) mask was generated from the first frame $I_{0}\in R^{1024\times 1280}$ . The image was first binarized (Equation 1) and subsequently processed using morphological hole filling and opening operations to obtain a clean binary mask representing the valid imaging domain. The image was first binarized and processed using morphological hole filling as described in Equation 2.

$$I_{fill}=\text{imfill}\left(\text{binarize}\left(I_{0}\right)\right)$$ (1)

Subsequently, morphological opening with a disk-shaped structuring element of radius 50 pixels was applied:

$$I_{open}=\text{opening}\left(I_{fill},\text{disk}\left(r=50\right)\right)$$ (2)

To eliminate unstable illumination bands, the top and bottom 10-pixel rows were excluded. The final FoV mask was defined as shown in Equation 3:

$$boundary\_img=I_{open}\cdot ud\_bar$$ (3)

**FIGURE 4:**
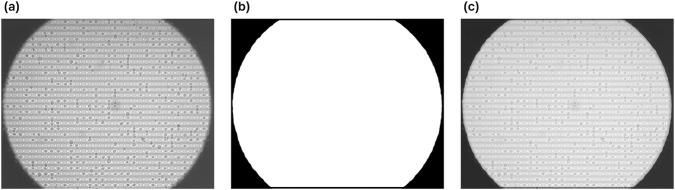
Boundary image generation process for defining out-of-range cells. **(a)** Original reference frame (first frame). **(b)** Generated boundary_img representing the valid observation area after morphological processing. **(c)** Alpha-blended visualization combining **(a,b)** Showing the overlay of the FoV mask on the original frame.

This mask was used to determine whether tracked cells remained within the valid field-of-view throughout the experiment (Figure 4).

#### 2.3.2 Deep learning-based object initialization

Cell detection was performed using a YOLOv8-based anchor-free convolutional neural network (Yaseen, 2024). For each detected cell at frame t , the detection state was represented as shown in Equation 4:

$$d_{t}^{i}=\left(x_{t}^{i},y_{t}^{i},r_{t}^{i}\right)$$ (4)

where $\left(x_{t}^{i},y_{t}^{i}\right)$ denotes the centroid coordinate and $r_{t}^{i}$ the estimated cell radius derived from the bounding box as defined in Equation 5:

$$r_{t}^{i}=\frac{w_{t}^{i}+h_{t}^{i}}{4}$$ (5)

Detected objects served as initialization points for trajectory association.

#### 2.3.3 Detection of DEP-induced rapid motion

Frequency switching between positive DEP (pDEP) and negative DEP (nDEP) induces abrupt cell displacement (Kwizera et al., 2021). To identify such transitions, the frame-wise average displacement was computed as shown in Equation 6:

$$\Delta_{t}=\frac{1}{N_{t}}\sum_{i=1}^{N_{t}}{∥\left(x_{t+1}^{i},y_{t+1}^{i}\right)-\left(x_{t}^{i},y_{t}^{i}\right)∥}_{2}$$ (6)

where $N_{t}$ is the number of detected cells at frame t . Rapid-motion frames were defined as local maxima satisfying the condition in Equation 7:

$$\Delta_{t}\geq \lambda \cdot \underset{k}{\max}\left(\Delta_{k}\right)$$ (7)

#### 2.3.4 Motion-aware adaptive ROI tracking

**FIGURE 5:**
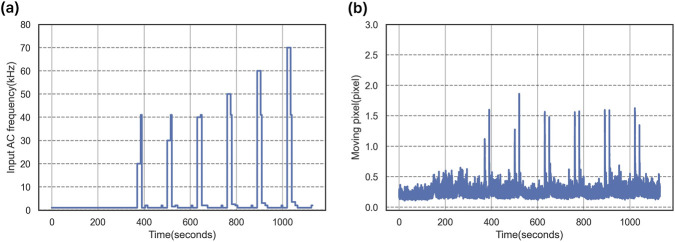
Frequency-modulated DEP stimulation and identification of rapid cell displacement events for adaptive ROI scaling. **(a)** The input AC frequency profile applied to the DEP chip over time. The experiment involves a series of frequency modulation steps that induce dielectrophoretic responses in living cells. **(b)** The average trajectory shift (displacement) of all detected cells per frame. Each red inverted triangle denotes a local maximum, corresponding to a sharp increase in cellular movement caused by DEP frequency switching. These peaks are used to define fast-movement intervals for dynamic ROI resizing.

To identify abrupt DEP-induced motion transitions, the frame-wise average displacement of all detected cells was computed. Rapid-motion frames were defined as local maxima of the average displacement curve exceeding the adaptive threshold described in Section 2.3.3. The frequency-modulated DEP stimulation profile and the corresponding trajectory displacement are illustrated in Figure 5. As shown in Figure 5a, the input AC frequency was periodically modulated, inducing polarity transitions in DEP force. The resulting cellular motion is reflected in Figure 5b, where the average per-frame displacement exhibits sharp peaks synchronized with frequency switching events. Each red inverted triangle marks a local maximum, corresponding to rapid DEP-induced movement intervals. These identified peaks were used to define fast-movement phases, during which adaptive ROI scaling was applied in the subsequent tracking stage. Tracking was performed independently for each detected cell using distance-based association. For a query cell q at frame t , the candidate match at frame was selected by minimizing the Euclidean distance, as defined in Equation 8:

$$\hat{i}=\arg\underset{i}{\min}{∥\left(x_{t+1}^{i},y_{t+1}^{i}\right)-\left(x_{t}^{q},y_{t}^{q}\right)∥}_{2}$$ (8)

Tracking parameters were adaptively adjusted depending on the motion regime. During rapid-motion intervals, a larger ROI radius and relaxed displacement and intersection-over-union (IoU) thresholds were applied. During stable-motion intervals, a smaller ROI and stricter thresholds were used to reduce identity ambiguity.

If no valid match was identified in the immediate subsequent frame, a local ROI centered at the previous centroid was defined, and candidate detections were searched within up to 10 subsequent frames. The nearest detection within the ROI was selected. If no detection was found, linear interpolation was applied to preserve trajectory continuity. Bounding circle–based IoU validation was additionally performed as shown in Equation 9:

$$IoU=\frac{A_{inter}}{A_{union}}$$ (9)

where $A_{inter}$ and $A_{union}$ denote intersection and union areas, respectively. Only matches satisfying $IoU\geq \tau_{IoU}$ were accepted.

#### 2.3.5 Out-of-range filtering and identity validation

After trajectory reconstruction, both temporal completeness and spatial validity of each trajectory were evaluated. To ensure consistent identity assignment throughout the sequence, tracking was restricted to cells detected in the first frame (t_0_). Objects entering the microscopy field of view after initialization were not assigned new identities and were excluded from trajectory reconstruction. This constraint prevents externally entering cells from confounding long-term trajectory analysis and $f_{co}$ estimation. A trajectory was classified as out-of-range if the condition in Equation 10 is satisfied:

$$|T_{q}|<T$$ (10)

where T denotes the total number of frames in the sequence. Because the experiment was conducted under continuous frequency modulation without intentional cell removal, a trajectory shorter than the full sequence length indicates that the corresponding cell existed the valid observation region during acquisition. To avoid misclassifying temporary tracking gaps as out-of-range events, multi-frame candidate search (up to 10 subsequent frames) and linear interpolation were performed prior to filtering. Therefore, trajectories were excluded only when cells physically left the observable field rather than due to transient detection or association failures. Spatial validity was determined using the predefined FoV mask (Figure 4) described in Section 2.3.1. When a trajectory centroid crossed the mask boundary, the corresponding cell was considered to have exited the field of view and was excluded from subsequent quantitative analyses, including $f_{co}$ estimation. This filtering step ensures that statistical analysis is performed only on trajectories with complete temporal coverage and spatial validity, thereby preventing bias introduced by truncated motion records. In addition, overlap-based identity validation was conducted to resolve rare merging or prolonged proximity events. When two trajectories exhibited excessive spatial overlap beyond the predefined IoU threshold for multiple consecutive frames, identity consistency was re-evaluated to prevent erroneous identity switching. This post-processing step further enhances trajectory reliability under high-density DEP conditions.

### 2.4 Determination of DEP crossover frequency from imaging-based analysis

To quantitatively evaluate cell-specific responses to frequency-modulated DEP, crossover frequency ( $f_{co}$ ) values were determined from the reconstructed single-cell trajectories obtained using DEP-Track. The $f_{co}$ represents the transition frequency corresponding to the effective zero-crossing of the DEP force between pDEP and nDEP, as inferred from observable changes in cell motion or trapping behavior (Choi et al., 2018; Choi et al., 2020; Park et al., 2020). In this study, the applied frequency range (1–41 kHz) resulted in a single observable transition between pDEP and nDEP, corresponding to a single crossover frequency for each cell. These motion-based analyses are grounded in the physical relationship between DEP-induced force and cell motion, where changes in motion direction directly reflect the sign of the Clausius–Mossotti factor. Two complementary image-based approaches were employed to estimate $f_{co}$ : (1) trapped image intensity analysis and (2) DEP force-induced velocity analysis.

#### 2.4.1 Trapped image intensity–based DEP crossover frequency determination

**FIGURE 6:**
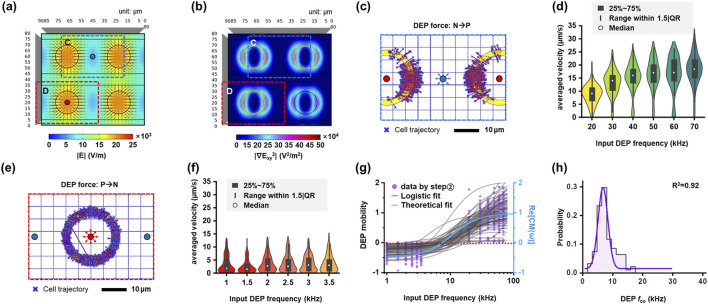
Workflow for DEP crossover frequency ( $f_{co}$ ) determination using velocity-based analysis. **(a)** Normalized electric field intensity distribution at a height of 9.5 μm above the electrode surface. **(b)** Gradient of the squared electric field intensity ( $\nabla |E_{\text{rms}}{|}^{2}$ ) obtained from COMSOL Multiphysics simulations. **(c,e)** Representative cell trajectories near the edge of the etched circular electrode under static DEP frequency conditions, showing displacement induced by polarity switching between negative DEP (nDEP) and positive DEP (pDEP). **(d,f)** Corresponding average velocity distributions presented as violin plots. **(g)** DEP mobility plot showing the normalized velocity term ( $v_{\text{cell}}/r_{\text{cell}}^{2}$ ) as a function of frequency. A sigmoidal logistic regression model was applied to determine the zero-crossing point corresponding to $f_{co}$ . The theoretical curve (blue line) was calculated using a single-shell DEP model for a 19 μm MCF-7 cell. **(h)** Histogram of the calculated $f_{co}$ values for MCF-7 cells derived from the fitted logistic model.

This intensity-based approach provides an empirical estimation of $f_{co}$ based on observable motion transitions, without requiring explicit modeling of DEP forces. The surface of the DEP microfluidic chip contains regions of local positive and negative divergence in the gradient of the squared electric field intensity ( $\nabla |E_{\text{rms}}{|}^{2}$ ), as illustrated by representative high-field and low-field points (red and blue markers) in Figures 6a,b. Under pDEP conditions, cells are attracted toward high-field regions (e.g., red markers in Figure 6a), whereas under nDEP conditions, cells migrate toward low-field regions (e.g., blue markers in Figure 6a) (Vaillier et al., 2016). In our experimental configuration, cells were initially trapped at convergent positions corresponding to either pDEP (e.g., 1 kHz) or nDEP (e.g., 41 kHz) static input frequencies. The trapped position of each cell was estimated using a Mahalanobis distance–based clustering approach applied to the trajectory under static frequency conditions (Choi et al., 2020; Park et al., 2020). During continuous frequency modulation (1–41 kHz, ±800 Hz/s), the image brightness intensity at the trapped location was monitored. The experimental $f_{co}$ was defined as the frequency at which a sudden deviation in image intensity occurred, consistent with previously reported definitions of crossover frequency. Specifically, $f_{co}$ was identified when the intensity exceeded three times the standard deviation of the baseline trapped intensity, indicating abrupt motion away from the trapping position. The electric field distributions, representative trapping locations, trajectory evolution, and intensity-based crossover detection are summarized in Figures 6a–g.

#### 2.4.2 Velocity-based DEP crossover frequency determination

According to DEP theory, the real part of the Clausius–Mossotti factor can be expressed as shown in Equation 11, a function of the balance between the dielectrophoretic force and the hydrodynamic drag force acting on the cell (Vaillier et al., 2016). When a cell moves in a region with a constant electric field gradient, the observed cell velocity reflects this force balance, allowing indirect estimation of the crossover frequency.

$$\text{Re}\left[CM\left(\omega \right)\right]=\frac{3\eta v_{\text{cell}}}{r_{\text{cell}}^{2}\varepsilon_{\text{medium}}\nabla |E_{\text{rms}}{|}^{2}}$$ (11)

where η is the medium viscosity, $v_{\text{cell}}$ is the cell velocity, $r_{cell}$ is the cell radius, $\varepsilon_{\text{medium}}$ is the permittivity of the medium, and $\nabla |E_{\text{rms}}{|}^{2}$ is the gradient of the squared electric field intensity. When cells move within regions where $\nabla |E_{\text{rms}}{|}^{2}$ is approximately constant, $\text{Re}\left[CM\left(\omega \right)\right]$ becomes linearly proportional as described in Equation 12:

$$\text{Re}\left[CM\left(\omega \right)\right]=k\left(\frac{v_{\text{cell}}}{r_{\text{cell}}^{2}}\right)$$ (12)

Importantly, the sign of $\text{Re}\left[CM\left(\omega \right)\right]$ determines the direction of DEP-induced motion, where positive values correspond to pDEP and negative values correspond to nDEP. Therefore, the $f_{co}$ is defined as the frequency at which $\text{Re}\left[CM\left(\omega \right)\right]=0$ , corresponding to a transition in the direction of cell motion. The electric field distribution and $\nabla |E_{\text{rms}}{|}^{2}$ were simulated using COMSOL Multiphysics to define annular regions adjacent to the circular electrodes for velocity analysis. For each input frequency, cell velocity was calculated from the travel distance and transit time of cells crossing the same ring region, allowing inward and outward motions to be compared within the same region (Figures 6a,b). Based on these simulations, ring regions with nearly constant $\nabla |E_{\text{rms}}{|}^{2}$ near the circular electrode structures were selected (Figures 6c,e). Trajectories of cells passing through these regions were extracted from the tracking results, and average velocities were computed at different input frequencies (Figures 6d,f). The normalized velocity term ( $v_{\text{cell}}$ / $r_{\text{cell}}^{2}$ ) was plotted as a function of frequency and fitted to determine the frequency at which $\text{Re}\left[CM\left(\omega \right)\right]=0$ (Figure 6g). The zero-crossing point was defined as the velocity-based $f_{co}$ for each cell. The resulting $f_{co}$ distributions were subsequently analyzed at the population level (Figure 6h). Representative electric field distributions, ring-region selections, and fitting results are provided in Figure 6. This velocity-based approach provides a physics-driven estimation of $f_{co}$ by explicitly linking cell motion to DEP force through the Clausius–Mossotti factor.

### 2.5 Evaluation protocol and performance metrics

To validate the robustness and scalability of DEP-Track under long-duration and frequency-modulated DEP conditions, a controlled comparative evaluation framework was designed. Three independent datasets (Dataset 1–3), each consisting of 13,200 frames acquired under identical frequency modulation protocols, were used for performance assessment. These datasets contained increasing cell densities to evaluate scalability under progressively challenging conditions. For fair comparison, three representative multi-object tracking frameworks—BoT-SORT (Aharon et al., 2022), ByteTrack (Zhang et al., 2022), and OC-SORT (Cao et al., 2023)—were evaluated. All methods were provided with identical cell detection outputs generated by the proposed YOLOv8-based detector (Chaurasia, 2023). This ensured that performance differences originated solely from trajectory association strategies.

#### 2.5.1 Detection performance metrics

Detection performance was evaluated using precision, recall, and F-measure. Let $C_{\text{GT}}$ denote the set of ground-truth cells and $C_{\text{pred}}$ the set of predicted cells. True positives ( TP ), false positives ( FP ), and false negatives ( FN ) were defined as shown in Equation 13:

$$TP=|C_{\text{pred}}\cap C_{\text{GT}}|,FP=|C_{\text{pred}}-TP|,FN=|C_{\text{GT}}-TP|$$ (13)

Precision and recall were computed as defined in Equation 14:

$$\text{Recall}=\frac{TP}{TP+FN},\text{Precision}=\frac{TP}{TP+FP}$$ (14)

The F−measure was calculated as shown in Equation 15:

$$F-measure=2\cdot \frac{\text{Precision}\cdot \text{Recall}}{\text{Precision}+\text{Recall}}$$ (15)

Ground-truth cell annotations were obtained via manual inspection of microscopy images.

#### 2.5.2 Tracking performance metrics

Tracking performance was evaluated using Multiple Object Tracking Accuracy (MOTA) (Bernardin and Stiefelhagen, 2008), as defined in Equation 16:

$$MOTA=1-\frac{\sum_{t}\left(FN+FP+{ID}_{sw}\right)}{\sum_{t}G_{t}}$$ (16)

where FN denotes missed detections, FP false positives, ${ID}_{sw}$ identity switches, and $G_{t}$ the number of valid ground-truth cells at frame t . Cells exiting the predefined observation region were excluded based on the FoV mask described in Section 2.3.1. In addition to MOTA, trajectory continuity analysis was performed by computing the fraction of successfully maintained trajectories over time relative to the initial number of tracked cells. This metric quantifies long-term identity preservation under dynamic DEP-induced motion conditions. Tracking evaluation was performed across three datasets with varying cell densities. A summary of the ground-truth cell counts used for MOTA computation is provided in Table 3.

**TABLE 3:** Summary of ground-truth cell counts for tracking evaluation.

Dataset	1^st^ detect	Out cells	GT cells
1	255	24	231
2	302	39	263
3	452	51	401

*1st detect indicates the number of cells detected in the first frame. *Out cells* represent the number of cells that moved out of the field during tracking. *GT, cells* refer to the number of cells used as the ground truth for tracking accuracy evaluation (denominator of MOTA).

### 2.6 Software implementation and computational environment

The proposed DEP-Track framework was primarily implemented in Python 3.8, with selected trajectory post-processing and crossover frequency ( $f_{co}$ ) analysis modules implemented in MATLAB R2024a (MathWorks, Natick, MA, USA). Detailed software and hardware specifications are provided in Supplementary.

## 3 Results and discussion

### 3.1 Cell detection performance on DEP microfluidic chips

**FIGURE 7:**
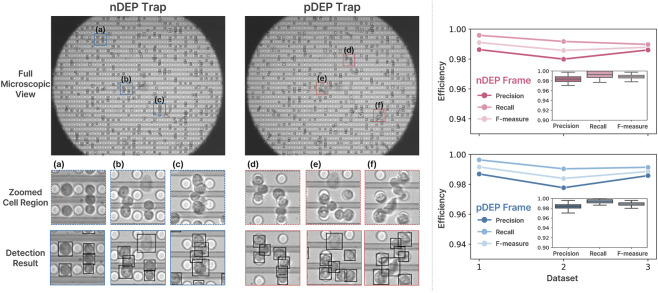
Cell detection performance under nDEP and pDEP trapping conditions. **(a–c)** Representative regions from the nDEP trap, including full microscopic view, zoomed cell regions, and corresponding detection results. **(d–f)** Representative regions from the pDEP trap, including full microscopic view, zoomed cell regions, and corresponding detection results.

**FIGURE 8:**
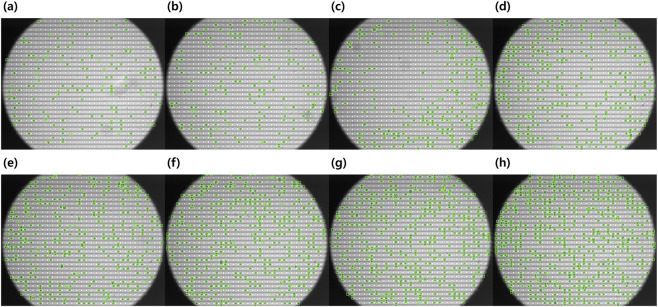
Detection results across eight DEP datasets with varying cell densities. Representative detection outputs for eight DEP sequences ranging from low to high cell density (138–564 cells per frame). Green bounding boxes indicate detected cells. **(a–h)** correspond to datasets with progressively increasing cell counts. The detector consistently achieves high accuracy even under crowded conditions.

Accurate cell detection on DEP microfluidic chips is inherently challenging due to structured electrode backgrounds, dynamic intensity variations, and frequent transient cell–cell adhesion events during DEP-driven motion. In densely populated chips, cells often partially overlap or temporarily attach to neighboring cells, requiring reliable discrimination of individual objects under crowded and dynamically changing imaging conditions. Representative detection results under positive and negative DEP trapping conditions are shown in Figure 7. Even in magnified regions containing closely positioned or partially overlapping cells, the proposed YOLO-based detector successfully localized individual cells without explicit preprocessing or background subtraction. These qualitative results demonstrate robustness against structured electrode patterns and complex optical interference characteristic of DEP microscopy. To determine the optimal detection backbone for the DEP-Track framework, an ablation study was conducted using five YOLOv8 variants (n, s, m, l, x) evaluated at two input resolutions (640 and 1,280 pixels). All models were trained and tested on the same DEP microscopy dataset, and inference time, GPU memory usage, and detection accuracy metrics (mAP50–95, mAP50, mAP75) were measured (Table 4). As summarized in Table 4, increasing model size and input resolution generally improved detection accuracy but required substantially higher memory consumption. Among all configurations, YOLOv8s with an input size of 1,280 achieved the best trade-off between detection accuracy and computational efficiency, reaching mAP50–95 = 0.7073, mAP50 = 0.9938, and mAP75 = 0.8864 while maintaining moderate memory usage (273 MB). Larger models (m, l, x) provided only marginal accuracy improvements relative to their computational cost, whereas smaller models (n) showed reduced robustness under dense imaging conditions. Based on this analysis, YOLOv8s (1,280) was selected as the detection backbone for DEP-Track. To further verify that the detection module does not limit overall pipeline performance, detection accuracy was evaluated across eight independent DEP datasets with varying cell densities ranging from 138 to 564 cells per frame (Figure 8; Table 5). For each dataset, true positives (TP) were computed as TP = GT − FN, where GT denotes the ground-truth cell count, FN false negatives, and FP false positives. Standard metrics including Precision, Recall, and F1-score were derived accordingly. Across all datasets, the detector achieved consistently high performance (Precision: 0.996–1.000, Recall: 0.982–1.000, F1-score: 0.989–1.000), even in sequences containing more than 500 cells per frame. These results indicate that detection accuracy remains stable as cell density increases and does not constitute a bottleneck in the DEP-Track framework. Error analysis revealed that occasional false positives were primarily caused by debris exhibiting unusually strong intensity that resembled cells. Rare false negatives typically occurred when cells were partially located near the boundary of the microscope field of view. In addition, burst or dead cells with extremely low contrast against the background were occasionally missed. However, these cases represented only a minimal fraction of the dataset and did not substantially affect overall detection reliability. To assess generalizability beyond MCF-7 cells, the trained detector was further evaluated on additional cell types, including A549, HeLa, and Jurkat cells (Supplementary Figure S1). Detection results under both nDEP and pDEP trapping conditions demonstrated consistent localization performance across different cell morphologies and DEP-induced response patterns. These findings suggest that the proposed detection module generalizes well to diverse DEP experimental settings and is not restricted to a single cell line.

**TABLE 4:** Ablation study of YOLOv8-based detection under different training configurations.

Detection model	Image size		Inference time (sec)	Memory (MB)	mAP50_95	mAP50	mAP75
	640	1,280					
yolov8n		✓	0.01508	64.44287	0.58561	0.952804	0.651845
	✓		0.02501	137.13671	0.676635	0.992482	0.82745
yolov8s		✓	0.02718	122.46826	0.643577	0.976506	0.765718
	✓		0.03726	273.11865	0.707306	0.993765	0.88644
yolov8m		✓	0.03197	303.37207	0.657055	0.98133	0.784609
	✓		0.03408	575.30224	0.71491	0.993771	0.882091
yolov8l		✓	0.03492	393.48583	0.643147	0.9782	0.754824
	✓		0.03000	837.66503	0.695186	0.993307	0.868559
yolov8x		✓	0.02156	952.41406	0.653667	0.977608	0.77945
	✓		0.04432	1013.5356	0.697874	0.993735	0.858381

**TABLE 5:** Detection performance across eight DEP datasets.

Case	GT (cell_num)	Detected	Precision	Recall	F1-score
(a)	138	137	1.00000	0.99275	0.99635
(b)	164	164	1.0000	1.00000	1.00000
(c)	238	239	0.99581	1.00000	0.99790
(d)	310	310	0.99677	0.99677	0.99677
(e)	330	328	1.00000	0.99394	0.99695
(f)	403	403	0.99504	0.99504	0.99504
(g)	454	454	0.99559	0.99559	0.99559
(h)	564	563	0.99822	0.99645	0.99733

TP (True Positives) is computed as the number of correctly detected cells (GT − FN), while FP (False Positives) and FN (False Negatives) correspond to non-cell detections and missed cells, respectively. Precision, Recall, and F1-score quantify the overall detection accuracy. Across all datasets, the detector achieves consistently high performance (Precision and Recall >0.99), confirming that cell detection is not a bottleneck in the DEP-Track pipeline. “Detected” represents the total number of cells predicted by the detector for each sequence.

### 3.2 Long-term tracking performance under frequency-modulated DEP

**FIGURE 9:**
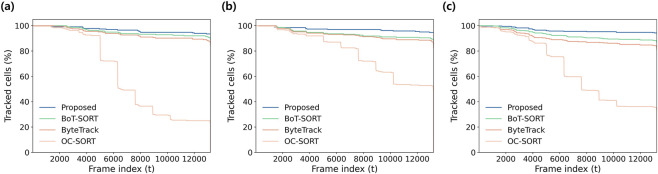
Tracking Continuity over frame. **(a)** Dataset 1, **(b)** dataset 2, and **(c)** dataset 3 show the percentage of cells that remained successfully tracked over frames for each DEP-Track (Y-axis represents the normalized percentage of cells that remained tracked over frames).

**FIGURE 10:**
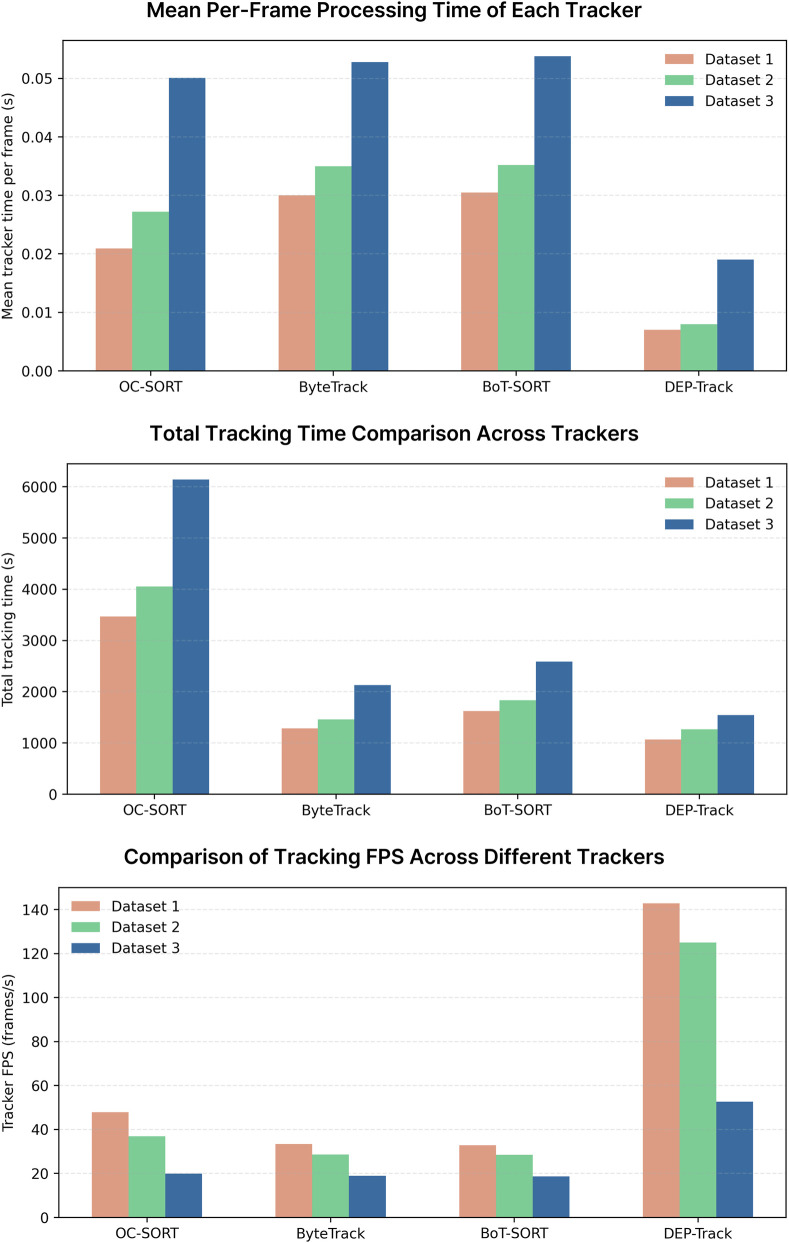
Runtime analysis across tracking algorithms and datasets.

Tracking performance was evaluated using three datasets, each consisting of 13,200 frames acquired under identical frequency-modulation protocols and progressively increasing cell density. Quantitative results are summarized in Table 6. Across all datasets, DEP-Track achieved the highest Multiple Object Tracking Accuracy (MOTA ≥93%) and maintained the largest number of successfully tracked trajectories while exhibiting the lowest loss rates. In addition to improved tracking accuracy, DEP-Track demonstrated substantially higher processing efficiency. In Dataset 1, the proposed framework reached 142.85 FPS, corresponding to approximately 3–4× higher throughput compared with baseline trackers. Normalized tracking retention curves are presented in Figure 9. DEP-Track preserved more than 90% of trajectories until the end of each sequence across all datasets, indicating stable long-term identity preservation over extended time-lapse recordings. In contrast, OC-SORT showed rapid decline in retention, particularly in Dataset 3 with the highest cell density. ByteTrack and BoT-SORT exhibited relatively stable but gradually decreasing retention curves as cell density increased, reflecting moderate robustness but progressive trajectory fragmentation. Further inspection revealed that tracking failures in baseline methods frequently occurred when cells moved in close proximity under DEP-induced forces, leading to temporary identity ambiguity. Under positive DEP conditions, some cells became trapped within electrode holes, where reduced contrast between the cell and chip background increased tracking difficulty. By incorporating motion-aware ROI adaptation, DEP-Track mitigated these issues and maintained more consistent trajectory association under dense and dynamically varying DEP conditions. Although most cells in the dataset exhibited near-spherical morphology, a subset displayed elongated, irregular, or deformed shapes. Representative challenging cases are summarized in Supplementary Figure S2. Even for elongated or elliptical cells, cells with blurred or partially indistinct boundaries, and shape-deformed cells under low-contrast conditions, DEP-Track maintained stable trajectory continuity. Representative tracking sequences illustrating these cases are provided as Supplementary GIF Files. These results indicate that the proposed framework is robust not only to high-density conditions but also to morphological variability and imaging degradation. Runtime performance analysis further confirmed the computational efficiency of DEP-Track (Figure 10). Mean per-frame processing time, total tracking time, and FPS were compared across OC-SORT, ByteTrack, BoT-SORT, and DEP-Track using identical detection inputs. Consistent with the quantitative measurements in Table 6, DEP-Track achieved the highest processing speed while maintaining superior tracking accuracy. In contrast, baseline trackers required longer processing time, particularly as dataset size and cell density increased. This balance between tracking robustness and computational efficiency supports scalable analysis of long-duration DEP microscopy sequences.

**TABLE 6:** Comparison of tracking performance.

Tracker	Dataset	Success Tracks ↑	MOTA(%) ↑	Total tracking Time (sec) ↓	Tracker FPS	DEP-track speedup
OC-SORT(Cao et al., 2023)	1	54	23.38	3470.2	47.84	**3.06× slower**
	2	132	50.19	4050.6	36.82	
	3	135	33.67	6141.3	19.96	
ByteTrack (Zhang et al., 2022)	1	207	89.61	1284.3	33.35	**3.96×** **slower**
	2	239	90.87	1456.6	28.55	
	3	345	86.03	2127.2	18.94	
BoT-SORT(Aharon et al., 2022)	1	211	91.34	1624.6	32.77	**4.02× slower**
	2	249	**94.68**	1835.5	28.41	
	3	362	90.27	2587.5	18.59	
DEP-Track (Proposed)	1	231	**93.51**	**1069.4**	**142.85**	—
	2	263	**94.68**	**1266.0**	**125.00**	
	3	401	**93.77**	**1543.8**	**52.63**	

*DEP-Track Speedup represents how many times slower each baseline tracker is compared to DEP-Track, based on their average FPS values. Bold values indicate the best performance.

### 3.3 DEP crossover frequency estimation using reconstructed trajectories

**FIGURE 11:**
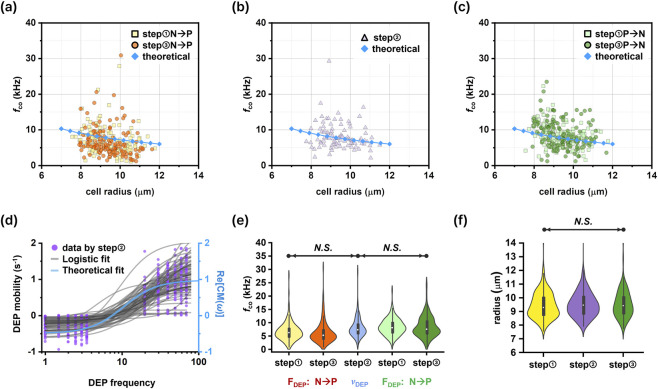
Representative crossover frequency ( $f_{co}$ ) distributions of MCF-7 cells determined using the dual $f_{co}$ estimation strategy. **(a–c)** Scatter plots of individual-cell $f_{co}$ values obtained under different DEP experimental steps, illustrating size-dependent variability. **(d–f)** Violin plots showing the distributions of $f_{co}$ and corresponding cell radius for the datasets presented in **(a–c)**. Cell radius was calculated as the average measured radius under the initial static-frequency condition for each experimental step. Violin plots include median and interquartile ranges. Statistical analysis was performed using one-way repeated-measures ANOVA followed by Bonferroni’s multiple-comparison test. The average $f_{co}$ and cell radius values were compared with previously reported measurements (Henslee et al., 2011; Park et al., 2020).

The crossover frequency ( $f_{co}$ ) distributions of living MCF-7 cells obtained using the DEP-Track framework are presented in Figure 11. Here, N → P denotes the polarity transition from negative to positive DEP, whereas P → N indicates the opposite transition. Using the velocity-based analysis described in Section 2.4.2, $f_{co}$ values were calculated under the static DEP frequency conditions defined in Step 2 of the experimental protocol. The resulting $f_{co}$ distributions are shown in Figure 11b. To visualize distribution characteristics, the raw data (Figures 11a–c) were represented using violin plots, which combine box plot statistics with kernel density estimation (Figures 11d–f). Statistical analysis was performed using one-way repeated measures ANOVA with Bonferroni’s multiple comparison correction. Across all tested conditions, no statistically significant differences were observed in either $f_{co}$ or cell radius distributions. The mean $f_{co}$ values remained consistent across polarity transitions and experimental steps. This consistency indicates that the trajectory-based estimation method provides stable and reproducible $f_{co}$ measurements, even under long-duration frequency-modulated DEP stimulation. Importantly, the average $f_{co}$ values obtained in this study showed good agreement with previously reported measurements for MCF-7 cells (Henslee et al., 2011; Park et al., 2020). This agreement supports the analytical validity of the DEP-Track framework and confirms that trajectory-derived velocity measurements can reliably capture polarity-dependent DEP transitions. These results provide DEP-specific validation of the proposed framework, as the estimated $f_{co}$ values are consistent with both previously reported measurements and statistically stable across experimental conditions. Beyond reproducing known $f_{co}$ values, the proposed framework enables population-scale characterization of DEP responses within a single experiment. Because individual cells are continuously tracked across multiple frequency cycles, DEP-Track allows extraction of full $f_{co}$ distributions rather than relying on repeated single-frequency measurements or manual observation. This distribution-level analysis facilitates assessment of intercellular variability and may provide insights into heterogeneous DEP-induced phenotypes, consistent with previous ensemble studies (Liu et al., 2020). This capability enables quantitative analysis of intercellular variability within nominally homogeneous populations. From a methodological perspective, the ability to estimate $f_{co}$ directly from reconstructed trajectories reduces dependence on manual identification of abrupt motion events. By combining trapping intensity–based analysis and velocity-based estimation within a unified computational pipeline, the framework ensures consistent identification of polarity transitions while maintaining scalability for high-throughput datasets. By integrating both trapping intensity–based and velocity-based analysis, the proposed framework provides complementary strategies for robust and consistent estimation of $f_{co}$ . Nevertheless, several considerations should be noted. The present study focused primarily on validating the robustness and reproducibility of $f_{co}$ estimation rather than performing detailed biophysical modeling of the complete DEP mobility spectrum. Moreover, although tracking robustness was demonstrated across dense imaging conditions, extreme occlusion or severe morphological deformation may affect trajectory continuity and consequently influence velocity-based estimation accuracy. Overall, these results demonstrate that DEP-Track provides a reliable and scalable computational approach for extracting $f_{co}$ information from long-term frequency-modulated DEP microscopy data. By enabling automated, population-level analysis of single-cell DEP behavior, the framework establishes a practical foundation for high-throughput dielectric phenotyping and future integration with physics-based DEP parameter modeling. From an application perspective, the ability to extract population-level $f_{co}$ distributions from long-term DEP measurements enables high-throughput, label-free analysis of cellular DEP responses. DEP-based techniques have been widely utilized for label-free cell phenotyping based on dielectric properties (Pethig, 2017), as well as for cancer cell discrimination in microfluidic systems (Gascoyne and Shim, 2014). In addition, previous studies have demonstrated the potential of DEP for parallel and high-throughput analysis of single-cell DEP responses (Liu et al., 2020).

## 4 Conclusion

In this study, we presented DEP-Track, an automated computational framework for scalable single-cell trajectory extraction and crossover frequency ( $f_{co}$ ) estimation from long-term, frequency-modulated DEP microscopy data. By integrating anchor-free cell detection with motion-aware trajectory association, the proposed method robustly preserves cell identity across tens of thousands of frames while simultaneously tracking large cell populations under dynamically changing electric field conditions. Using two complementary DEP analysis strategies—velocity-based analysis under fixed frequencies and trajectory-based analysis under continuous frequency modulation—DEP-Track consistently produced stable and statistically consistent $f_{co}$ distributions in living MCF-7 cells. The estimated $f_{co}$ values showed strong agreement with conventional workflows and previously reported measurements, confirming the accuracy and reliability of the proposed approach. Beyond numerical agreement, DEP-Track enables population-scale characterization of single-cell DEP responses within a single experiment by continuously tracking the same cells across multiple frequency cycles. This capability overcomes key limitations of traditional DEP analysis methods that rely on repeated measurements or manual post-processing, thereby enabling high-throughput and reproducible single-cell dielectric phenotyping based on crossover frequency ( $\left.f_{co}\right)$ . Despite these strengths, several limitations should be acknowledged. First, the detection model was primarily trained on round and oval-shaped MCF-7 cells, and its performance for cells with markedly different morphologies or imaging characteristics may require retraining and further validation. Second, tracking performance may decrease under complete occlusion scenarios in which multiple cells fully overlap, making individual identity recovery infeasible. Third, the present study focused on validating the computational framework and confirming the consistency of $f_{co}$ estimation, rather than exhaustively interpreting the full biophysical implications of the entire DEP mobility spectrum. In addition, although preliminary validation suggests applicability across multiple cell types, further validation using a broader range of primary cells and experimental conditions will be necessary to fully establish generalizability. Future studies will therefore expand the training dataset to encompass diverse cell types and experimental conditions, incorporate advanced re-identification or occlusion-handling strategies, and integrate physics-informed DEP models to enable quantitative fitting of single-cell dielectric parameters. Such extensions will transform DEP-Track from a robust tracking and $f_{co}$ extraction tool into a comprehensive platform for high-throughput, physics-informed dielectric phenotyping. Owing to its lightweight and computationally efficient design, the framework supports scalable analysis without excessive computational burden and can be readily extendable to other microelectrode-based DEP assays. Overall, DEP-Track establishes a practical and robust foundation for automated DEP analysis and provides a versatile platform for future biomedical applications, including high-throughput label-free cell phenotyping, drug-response profiling, and quantitative characterization of heterogeneous cell populations based on crossover frequency–derived DEP responses. These capabilities highlight the potential of DEP-Track as a scalable and practical tool for DEP-based biomedical analysis workflows.

## Data Availability

The raw data supporting the conclusions of this article will be made available by the authors, without undue reservation.
