# Supplementary material for: DEP-track: a motion-aware framework for large-scale cell tracking and crossover frequency estimation in dielectrophoresis
Source: Front Bioinform. 2026 Apr 23;6:1821804. doi: 10.3389/fbinf.2026.1821804 (PMC13149371; doi:10.3389/fbinf.2026.1821804)
Supplement: Supplementary file 1 [file Supplementaryfile1.docx]

Supplementary Material

Supplementary Figures


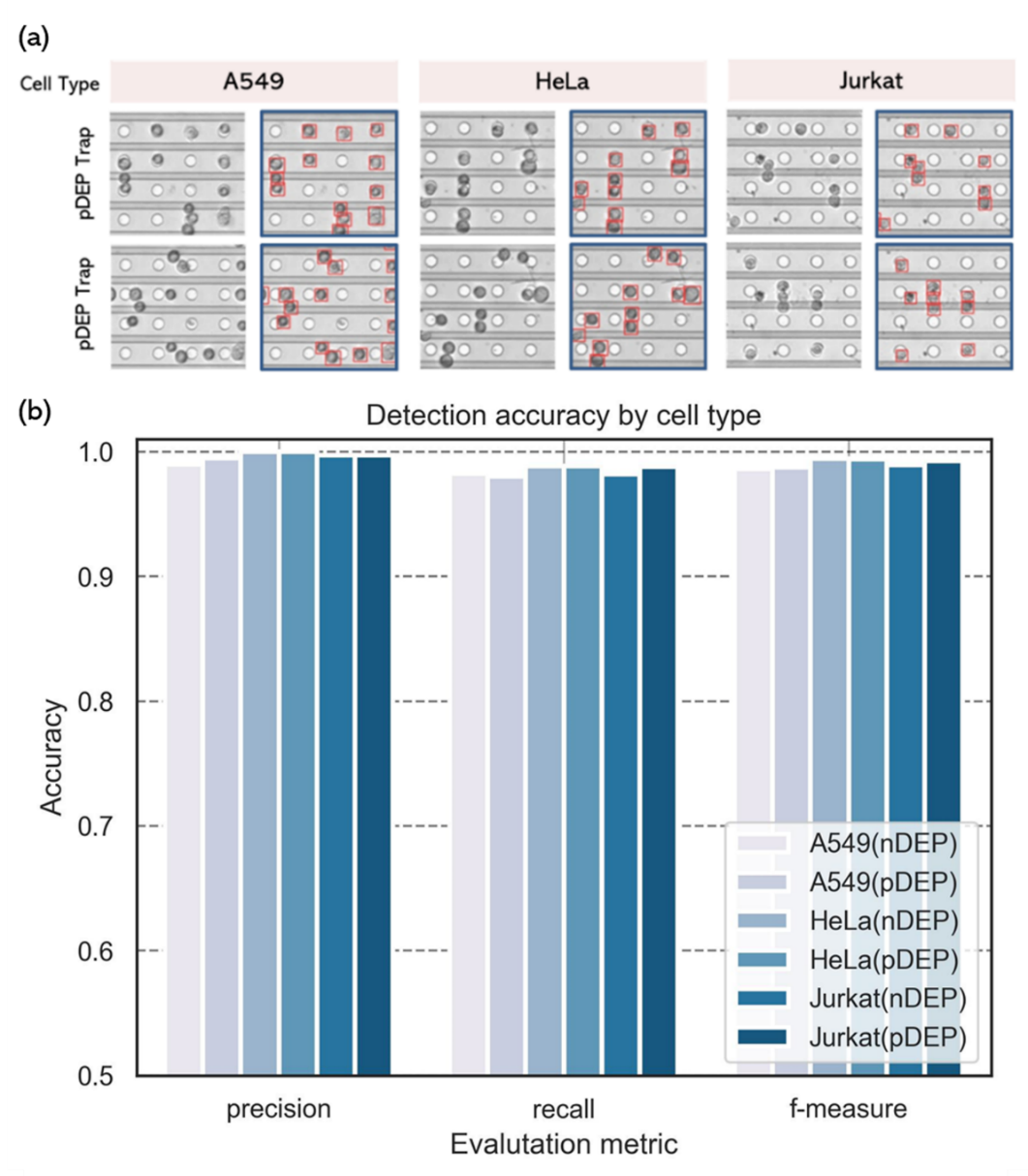


**Supplementary Figure S1.** Detection performance on additional cell types. (a) Representative detection outputs of the proposed system applied to different cell lines (A549, HeLa, and Jurkat). (b) Quantitative detection accuracy under both negative DEP (nDEP) and positive DEP (pDEP) trapping conditions.

| **Case** | **ROI image** | **Tracking Result(GIF)** |
| --- | --- | --- |
| Case1**. Elongated (Elliptical)  Cell Shapes**  Cells exhibiting elongated or elliptical morphology. | 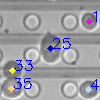 | 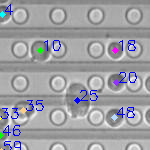 |
| Case2. **Cells with Blurred or  Faded Boundaries** Cases where the cell boundary becomes blurred or partially indistinct due to imaging conditions. | 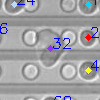 | 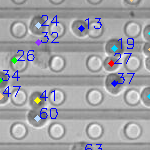 |
| Case3. **Shape-Deformed Cells Under Low Contrast** Cells whose shapes are deformed and whose contrast against the background severely decreases. | 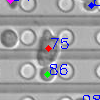 | 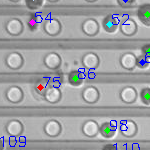 |

**Supplementary Figure S2.** Representative tracking performance of DEP-Track under morphologically challenging conditions. Representative tracking outcomes of the proposed DEP-Track framework applied to cells exhibiting elongated, irregular, or deformed morphologies. Although the majority of cells in our dataset display near-spherical shapes, a subset presents significant elongation or boundary irregularities. These examples demonstrate that the detector–tracker pipeline maintains reliable detection and identity preservation even when cell morphology becomes deformed, boundaries appear blurred, contrast is reduced, or partial overlap with electrode structures occurs. Corresponding animated GIF files illustrating the tracking sequences are provided as separate supplementary materials.

**Software Development Environment**

The performance of the proposed DEP-Track algorithm was evaluated using a long-term time-lapse microscopy sequence consisting of 13,200 frames with a spatial resolution of 1024 × 1280 pixels.

Detection experiments were performed on a workstation equipped with an AMD Ryzen 7 7800X3D 8-Core Processor (32 GB RAM, 3.20 GHz) and an NVIDIA GeForce RTX 4090 GPU. Additional benchmarking and validation experiments were conducted on a separate system with an Intel Core i7-8700K CPU (32 GB RAM, 3.20 GHz) to verify cross-platform consistency.

All deep learning–based detection processes were implemented in Python 3.8 using the PyTorch framework with GPU acceleration. The trajectory association and motion-based crossover frequency analysis modules were implemented in MATLAB R2024a (MathWorks, Natick, MA, USA). This hybrid implementation reflects the modular structure of the proposed framework and enables flexible integration with existing DEP analysis pipelines.
